# Effects of Nanomaterials on the Fresh and Hardened Properties of Concrete: A Review

**DOI:** 10.3390/nano16070426

**Published:** 2026-03-31

**Authors:** Gashaw Abebaw Adanu, Bolanle Deborah Ikotun, Rasheed Abdulwahab

**Affiliations:** Department of Civil & Environmental Engineering and Building Science, University of South Africa, Johannesburg 0002, South Africa; ikotubd@unisa.ac.za (B.D.I.); abdulr@unisa.ac.za (R.A.)

**Keywords:** concrete, nanomaterial, mechanical properties, concrete sustainability

## Abstract

Insufficient tensile strength, low abrasion resistance, and inadequate consistency in the fresh state led to fractures and decreased the durability of the concrete. Tensile stress resistance is the most challenging, resulting in the formation of microcracks that propagate to a macrolevel. Nanomaterials, with dimensions ranging from 0.1 to 100 nanometers, represent an innovative class of materials that can enhance the mechanical properties of concrete through the nano-core effect. These materials play significant roles in the formation of calcium–silicate–hydrate (C-S-H) gels, contribute to seeding effects, and augment cement hydration reactions. Given the above, the addition of nanomaterials makes concrete exhibit exceptional mechanical strength and improved durability performance. The primary objective of this review is to identify the potential nanomaterials suitable for the development of high-performance concrete. This article reviews the literature on the effects of nanoparticles, such as nano-calcium carbonates (NCCs), iron oxide (NI), nano-aluminum oxide (NA), graphene oxide (GO), nano-silica (NS), and nano-titanium oxide (NT) on the fresh and hardened properties of the material. The study identifies a promising nanomaterial for enhancing concrete, highlights research gaps, and suggests future research directions for its optimal application in future concrete constructions.

## 1. Introduction

Concrete is a commonly utilised material in the construction of infrastructure across the world, consisting mostly of cement and aggregate [1]. Concrete has weak tensile strength [2], poor abrasion resistance [3], and cracking issues [4], which require meticulous handling. The addition of nano-reinforcement to cement composites is a significant option because of its ability to increase mechanical strength, tensile strength, and durability properties [5,6]. Concrete reinforcement is made feasible by the synthetic characteristics and pore-filling ability of nanomaterials, which range in size from 1 to 100 nm [5]. The effects of nanoparticles on cement-based products have been the subject of several studies. Nanomaterials have been added to concrete to improve its properties. Their microscopic sizes facilitate an increase in the surface area, thus accelerating the hydration process and strengthening the concrete matrix on the nanoscale [7,8].

Future technical advancements in concrete engineering will be driven using nanomaterials, which have been shown to increase the mechanical strength and durability of concrete, according to research conducted by academics worldwide. Numerous researchers have examined the impact of various nanomaterials on the characteristics of concrete and discovered various improvements. Nano-calcium carbonate (NCC) increased the flexural strength at 28 days by 4% with a 1–6% addition [9], while another researcher discovered that 0.5% NCC significantly improved the concrete mechanical strength [10]. The addition of 3% nano-alumina (NA) improved the 28-day compressive strength by 1.07% [11], whereas 1% NA improved the 28-day compressive strength by 16% [12]. The incorporation of a 0.1% carbon nanotube (CNT) improved the compressive capacity by 31.8% [13], whereas a 0.5% CNT improved the compressive strength by 27.8% [14]. The addition of 0.3% nano-iron (NI) strengthened the 28-day flexural strength by 58.2% [15], while 2.5% NI improved it by 13% [16]. The addition of 0.03% graphene oxide (GO) increased the compressive strength by 28% [17], while 0.08% GO increased it by 21% [18]. The preceding narration declared that nanomaterials improve the mechanical properties of concrete, although there is a variance in the nanomaterial used and its influence on mechanical strength.

This study reviews the literature on the effects of nanomaterials (GO, CNT, NA, NI, NCC, NS, NT) on concrete properties. This study provides scientific suggestions for using nanomaterials in the production of concrete and recommends future research topics to evaluate the prospects of adding nanomaterials to concrete. This study reviews the literature on the effects of nanomaterials (GO, CNT, NA, NI, NCC, NS, NT) on concrete properties. The study is limited to the above nanomaterials only due to the breadth of the study areas. In the future, the author plans to address the remaining nanomaterial applications on concrete properties. Finally, the study identifies a promising nanomaterial for enhancing concrete, highlights research gaps, and suggests future research directions on incorporating nanomaterials into concrete.

## 2. Materials and Methods

### 2.1. Materials

This review paper conducted an in-depth examination of seven distinct nanomaterials concerning their applications in concrete. Each nanomaterial exhibits different properties owing to its chemical composition, surface area, size, and purity. To this, a CNT possesses a particle size diameter ranging from 20 to 100 nm, a surface area of 50 to 260 m^2^/g, a length of 5 to 15 mm, 3% amorphous carbon, and a purity of 94 to 99% [19,20,21,22]. In a similar manner, nano-TiO_2_ is characterized by an average particle size of 15 nm, a purity exceeding 99.00%, a surface area ranging from 40 to 250 m^2^/g, and a diameter of 5 to 20 nm [23,24,25]. The surface areas and respective particle sizes for the analyzed nanomaterials are as follows. NA exhibits a surface area range of 25 to 180 m^2^/g [26,27]; NCC features a surface area of 40 m^2^/g with a particle size spanning 15 to 60 nm [9,28]; NI presents a surface area exceeding 30 m^2^/g and particle sizes ranging from 20 to 200 nm [29,30]; GO is characterized by a surface area of 110 to 250 m^2^/g with particle sizes of 0.8 to 2 nm [31]; and NS displays a surface area of 300 g/m^2^, accompanied by particle sizes from 7 to 40 nm [20]. The chemical composition data pertaining to NA, NCC, NI, GO, and NS are readily available. However, there exists a significant paucity in the literature regarding the chemical composition details of nano-carbon nanotubes and titanium. Table 1 illustrates the chemical compositions of each material, which are described below.

### 2.2. Review Methods

**Figure 1:**
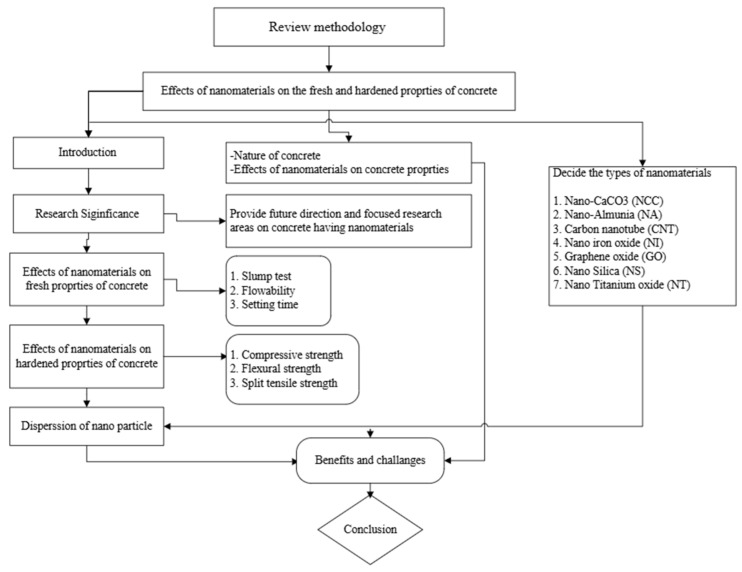
Schematic illustration of review methodology.

This article reviews recent studies on nanomaterial-containing concrete. The method used in this study is an in-depth examination of previous studies to arrange the results for future researchers on nanomaterial-containing concretes. The study intends to examine how nanomaterials affect workability, setting time, flowability, compressive strength, flexural strength, and split tensile strength of concrete, in addition to evaluating the mix percentage, curing time, and dispersion. The paper continues by showing areas for future research that may necessitate more substantial investigation into the usage of nanomaterials in mixtures of concrete. Figure 1 depicts the flowchart used to review nanomaterial inclusion in concrete.

## 3. Results

### 3.1. Effect of Nanomaterials on the Fresh Properties of Concrete

The fresh properties of concrete have been affected by the interaction of nanomaterial particles. The effects of carbon nanotubes, graphene oxide, nano-iron oxide, nano-silica, nano-CaCO_3_, and nano-titanium in the production of concrete were extensively examined in this study.

#### 3.1.1. Nano-CaCO_3_ (NCC)

Calcium carbonate (CaCO_3_) has been a good nanomaterial (NM) in the development of cement composites [32,35]. It improves heat of hydration, decreases flowability [36], increases early strength development [37], increases the mechanical strength of concrete [36,38], and reduces cracks due to creep and shrinkage, having the potential of reducing the concentration of stresses being developed in the composite [28]. The wide usage of calcium carbonate by different industries is on the increase because it is a non-toxic, environmentally benign mineral with a large surface area and posity in both nano- and microindustries [39].

Ref. [39] examined the effects of NCC on the fresh properties of concrete and found that concrete with calcium carbonate nanomaterials had a longer setting time than the control mix. The incorporation of NCC reduces the workability of the concrete. According to research by [40], adding 2 and 3% NCC resulted in a 13 mm slump loss. The addition of 1% NCC affects concrete workability more than silica fume addition, yet NCC has a larger surface area. Ref. [9] examined the effects of NCC on foam-based concretes. The flowability of the control mixture was 259 mm, whereas the addition of 6% NCC decreased the flowability to 238 mm. Compared to the control mixture, the addition of NCC in the range of 1–6% reduced workability by 1.2–8.1% [9].

The addition of 4% NCC increased the amount of superplasticizer, increasing from 2.0% in the plain mixture to 2.5% in 0.5% carbon fiber. The integration of nano-calcium carbonate into concrete increases the amount of water required for the mixing and hydration, making CaCO3 a problematic nanomaterial for concrete production [39]. The addition of 4% nano-calcium carbonate increased the superplasticizer content from 2.0% in the plain mixture to 2.5% in 0.5% carbon fiber [41]. NCC additions of up to 5% improved the flowability of concrete by 30%, whereas concrete workability was hampered by replacements exceeding 5–15% by 54% [41]. Research by [41] on the effect of NCC on the ultra-high-performance of concrete concluded that NCC improved the early age development of concrete cured at 10–20 °C.

#### 3.1.2. Nano-Alumina (NA)

Nano-alumina is another nanomaterial used in concrete that improves mechanical strength, frost resistance, freezing and thawing resistance, and concrete durability [42,43]. A more compact microstructure led to a significant improvement in the frost resistance of concrete containing nano-alumina particles [44]. Since nano-alumina consumes the CH generated by the hydration reaction, it reduces the holes and cracks in the concrete structure, improving the development of C-S-H gels [11]. Ref. [37] examined the effect of a concentration of 1–2% non-aluminum in a concrete mixture having 10–20% metakaolin. The inclusion of NA improved the workability of concrete, with the control mix having a slump value of 65 mm increasing to 92 mm, 95 mm, and 98 mm with the addition of 1, 1.5, and 2% NA, respectively [37]. The inclusion of NA significantly affects the workability of concrete, and according to [26], the water requirement was found to increase as the level of NA increased [45] investigated the influence of NA on the workability of ultra-high-performance concrete. The flowability of the control mix was 98%; however, the addition of NA increased the flowability to 110% [46]. According to the data mentioned previously, there are doubts about the effects of adding NA on the properties of freshly mixed concrete, and further investigation is required.

#### 3.1.3. Carbon Nanotube (CNT)

CNTs are hollow two-dimensional tubes that are incredibly strong and thermally efficient [47] and improve the mechanical strength of concrete at high temperatures [48,49]. In the concrete production process, CNT improves the cement hydration process, improves the filling of pores, and eliminates cracks in the concrete substrate [50]. Ref. [51] investigated the effect of CNT on the workability of steel fiber-reinforced concrete and found that concrete containing 0.15% CNT decreased workability by 29.89%. CNTs have strong bonding capabilities with concrete materials and large surface areas, which reduce their workability [51,52]. CNTs considerably affect the workability of concrete; the addition of 0.1 and 0.3% CNT reduced the workability of lightweight concrete by 17.7 and 27.5%, respectively [21].

#### 3.1.4. Nano-Iron Oxide (NI)

Refs. [30,53] investigated the effect of NI on the performance of fly ash blended mortar and revealed that the addition of NI increased the workability of concrete. Compared to the control mix, the addition of 15% NI increased the flowability of the mortar by 12.5% [30]. Ref. [33] discovered that the addition of NI reduced the slump of concrete, and the control sample had a slump value of 40 mm; this value was reduced to 16 mm by adding 5% NI [33]. Concrete workability decreased as the amount of NI increased, with a correlation value of 0.9439. The addition of NI can increase the setting time of mixed fly ash concrete, with an initial setting time of 75 min and an ultimate setting time of 210 min [54]. The addition of different amounts of NI improved the flowability of concrete; the flowability of the control mix was 245 mm, whereas the addition of 0.10% and 0.35% NI improved the flowability to 253 mm and 278 mm, respectively [55]. Different researchers have reported contradictory results on the effect of NI on the fresh properties of concrete. Further investigation is needed to determine the effect of NI on the properties of cement composites in the fresh state.

#### 3.1.5. Graphene Oxide (GO)

The graphene derivative of GO is composed of one layer of sp2-hybridized molecules that have undergone functionalities such as carboxyl, hydroxyl, or epoxy modifications [56,57]. The high-water solubility of graphene oxide in water makes it a promising material for concrete manufacturing. The modified Hammers technique was used to synthesize graphene oxide, and the X-ray diffraction results showed that the peak diffraction occurred at 2θ = 122.02°, and the graphite peak was detected at 2θ =26.62° [58]. The fundamental cause of the observed peak was the existence of a reliable structure. Two peaks for GO were seen at 9.03°, showing that the graphite had been completely oxidized to GO. This confirms that GO and reduced graphene oxide (rGO) were effectively synthesized [59]. Ref. [18] investigated the effect of GO on the workability of fresh concrete and found that the addition of GO decreased the workability of concrete. The addition of GO influences the concrete hardening time; the setting of the control sample was 330 min, and the addition of 0.01% GO reduced the set time to 300 min [17]. GO is a tiny nanoparticle with a large surface area that reduces the workability of concrete by increasing the water requirement for optimal workability [18,34,60]. GO significantly decreases the workability of concrete and improves the setting times of fresh concrete.

#### 3.1.6. Nano-Silica (NS)

The concrete mix’s flowability and workability were decreased by the addition of NS [61,62,63]. Ref. [63] tested the influence of NS on the workability of fly ash blended concrete and discovered that adding NS decreased the workability of the concrete. The addition of 5, 10, and 15% NS resulted in flowability of 105, 99 mm, and 93 mm, respectively. This trend shows that the addition of NS decreased the flowability of the concrete when compared to the control mix, which had a value of 110 mm [63]. The addition of 0.5, 1, and 1.5% NS significantly decreased the slump of high-performance concrete (HPC). The slump value of the control mix was 46.5 cm, whereas adding NS at 0.5, 1, and 1.5% resulted in 39.5, 33.5, and 27.5 cm, respectively [61]. The workbility of high-strength concrete was reduced when NS was added; the control mix had a slump of 112 mm, while the slump values of the concrete with NS added at 1, 3, and 5% were 109, 104, and 100, respectively. Properties of sustainable high-strength concrete containing large quantities of industrial wastes, nanosilica and recycled aggregates [64]. NS reduces the workability of concrete because of its large surface area, swift hardening, and increased viscosity [61].

#### 3.1.7. Nano-Titanium Oxide (NT)

The inclusion of NT considerably lowered the sump and flowabiity of the concrete, which contributed to its lower workability [65,66]. The inclusion of 0.5, 1, and 1.5% NT to waste glass blended concrete lowered the slump of the concrete by 4.76, 12.38, and 16.19%, respectively, as compared to the control mix. Hence, the addition of NT significantly reduced the workability of the concrete [67]. The control mix for M40 concrete had a slump value of 44 mm; adding NT at concentrations of 0.5, 1, 1.5, and 2% resulted in slump values of 35 mm, 32 mm, 26 mm, and 21 mm; the control mix for M60 concrete had a 37 mm slump, while the additions of 0.5, 1, 1.5, and 2% produced slumps of 33, 27, 24, and 18 mm, respectively. Taking all of this into account, the addition of NT significantly decreased the workability of the concrete [68]. The flowability of self-compacting concrete reduced as the amount of NT increased [69]. The workability of concrete reduced as the NT content grew from 0.5 to 3%. This is due to NT’s low water content, insufficient lubricating behavior, and quick rate of water absorption [70]. Numerous studies have found conflicting findings about the influence of NT on the fresh characteristics of concrete. Further research needs to be conducted to affirm the influence of NT on the fresh characteristics of the concrete.

### 3.2. Effect of Nanomaterials on the Hardened Properties of Concrete

Concrete’s flexural strength, split tensile strength, compressive strength, and other hardened concrete characteristics have all been affected by the interaction of nanomaterial particles. Nano-CaCO_3_, nano-aluminum, nano-iron oxide, nano-silica, graphene oxide, nano-titanium, and carbon nanotubes were all considered thoroughly in this paper.

#### 3.2.1. Nano-CaCO_3_ (NCC)

The effects of NCC on the mechanical qualities of foamed concrete keeping the cement-to-aggregate ratio at 1:1.5 and the water-to-binder ratio at 0.45. Discovered that the addition of NCC increased flexural, compressive, and split tensile strength abilities [9]. The concentrations of NCC ranged from 0 (control) to 6%, and all test protocols followed ASTM standards. On the 28th day of flexural strength testing, the addition of NCC in the range of 1–6% was explored; the results indicated that the flexural strength of foamed concrete improved by up to 4%, while a decrease was observed at 5 and 6% addition [10]. Ref. [10] investigated the effect of NCC concentration at 0.5, 0.7, and 1% on the hardened characteristics of concrete blending 80 kg/m^3^ fly ash. Fly ash blended concrete with 0.5% nano-CaCO_3_ showed higher 7- and 28-day flexural strengths than the control mix, which could be attributed to the high silicate content of fly ash, which interacts with CaCO_3_ to form additional hydration products of the CSH bond [10]. The elastic modulus of concrete increased by 5–12% after 28 days when 1–3% NCC was added [28]. The study opined that NCC improved the formation of the C-S-H gel, which could be due to the seeding effect produced by NCC [28,71,72].

Ref. [71] investigated the effect of NCC at 1, 2, 3, and 4% on the characteristics of high-volume fly ash (40 and 60%) concrete. They discovered that the addition of NCC initially increased the mechanical strength of the concrete but weakened as the amount of NCC increased. At 7 and 28 days, the combination of 1% nano-CaCO_3_ ash increased the compressive strength by 22 and 18%, respectively. The early strength of concrete containing fly ash was found to be poor, and the strength development declined as the amount of fly ash increased [73,74]. The addition of NCC to mixed fly ash concrete improves the development of its compressive strength [71], allowing an increase in the amount of fly ash replacement without compromising the mechanical strength of mortar and concrete. However, injecting 1% NCC into mortar having 40–60% fly ash increased its compressive strength. After seven days, the compressive strength increased by 21% with 40% FA [71]. The compressive strength at 7 and 28 days was enhanced by adding 1% NCC, and the compressive strength at the early age of 7 days was the highest, with a 22% improvement over the reference mix, and at 28 days, it increased by 18% [71]. A similar study by [32] found that the addition of NCC increased compressive strength. Ref. [38] found that adding NCC to fiber-reinforced concrete increased its mechanical strength. The presence of NCC increased the compressive strength of fiber-reinforced concrete to 120 MPa, exceeding the required standard for high-performance concrete. The compressive strength of concrete containing 0.5% carbon fiber without NCC increased steadily by 1.2% [38].

**Figure 2:**
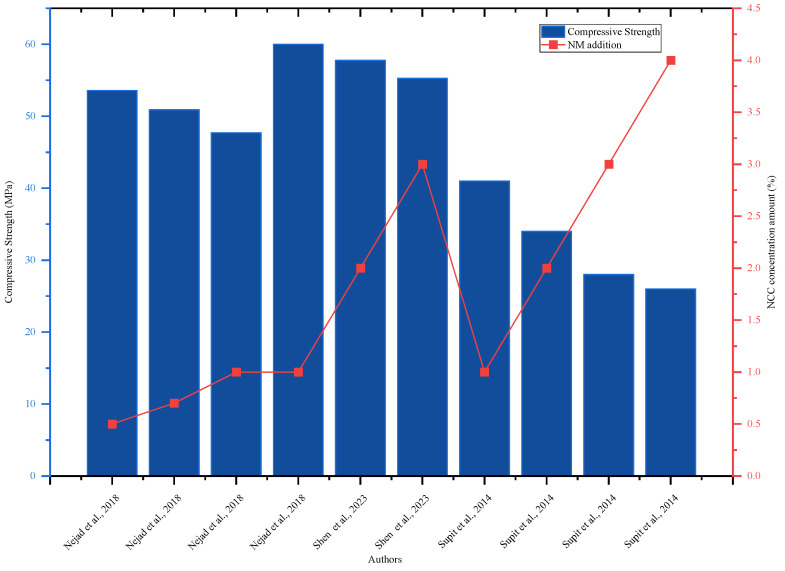
Effect of NCC on 28-day compressive strength, highlighting strength improvement with increasing NCC content by referring to different authors’ data [10,28,71]. A scanning electron microscopy (SEM) image analysis of the NCC’s impact on the internal structure of FA blended concrete is presented in Figure 3, which reveals that the inclusion of NCC increased the internal structure’s density and reduced its porosity [10].

Ref. [9] investigated the influence of NCC on the tensile strength qualities of the split concrete foam. The greatest improvement in split tensile strength was seen after 7 and 28 days with a 4% addition of NCC, which indicated a 52% increase over the reference mix. Researchers suggested that incorporating nanofibers into concrete would be the best approach to minimize their risk of rupture. Research by [10] investigated the effects of NCC on the tensile properties of fly ash blended concrete and found that the tensile strength of the split improved in 7- and 28-day tests. The addition of 0.5% NCC increased the tensile strength of the split to 26.50 MPa and 24.80 MPa compared to mixed concrete with FA, which had a tensile strength of 15.80 MPa and 12.60 MPa [10]. Regarding Figure 2, the addition of NCC increased the concrete’s compressive strength until it reached the ideal level of 3%, after which it decreased as the concentration increased. Based on concrete performance evaluation, the optimal NCC concentration is 3%, which meets the best quality of concrete.

#### 3.2.2. Nano-Alumina (NA)

Ref. [11] investigated the effect of NA addition at 1, 2, 3, and 4% of cement on the mechanical characteristics of concrete while maintaining a water-to-binder ratio of 0.4 and discovered that adding NA to the concrete mix enhanced its mechanical qualities up to the optimal replacement level; the curing was in a low temperature at 5 ± 0.2 °C. All the samples were prepared and cured in an open tank at 5 ± 0.2 °C for curing. The addition of 1, 2, and 3% NA affected the compressive strength; 3% NA increased normal concrete by 1.09, 1.02, 1.01, and 1.07%, respectively, for each day of 3, 7, 14, and 28 days [11]. According to [53], up to 2% NA enhanced the compressive strength of M55-graded concrete, while additions of 2–3% could have deteriorating effects on the concrete. According to the investigation by [11] the maximum compressive strength was found with 1% NA addition, which improved compressive strength by 3, 7, 14 and 28 days by 42, 25, 17, and 16%, respectively, as compared to the reference mix. According to [12] the addition of 1% NA increased the compressive strength of the M40 grade concrete by 42.36, 35.88, and 14.41% after 7, 14, and 28 days of evaluation, respectively. The addition of NA has a significant impact on the compressive strength of the metakaolin blended concrete. Ref. [62] investigated the influence of NA addition on the mechanical strength of mixed metakaolin (MK) concrete and discovered that with 1–2% NA inclusion in the concrete and partial substitution of cement with MK with 10- 20%, the compressive strength was greater than the control mix. The addition of NA improved compressive strength; a maximum compressive strength of 38.4 MPa was recorded.

The research by [75] explored the effect of NA at 0.02, 0.75, 1, 3, and 6% on geopolymer concrete made with natural laterite soil as a filler material. The concrete performed significantly more effectively when NA was added. After 7 days, 0.75% of the concrete had 80% compressive strength. The inclusion of 2% NA improved the flexural strength of geopolymer concrete after 7 and 28 days by 35.54 and 53.76%, respectively [76]. According to [12], adding 1% NA to the concrete increased the tensile and flexural strength of the flexural and split, while decreasing it by more than 1% NA. Concrete compressive strength was tested after adding 1, 2, 3, and 4% NA at 7, 14, and 28 days. The findings showed that the addition of 1% NA significantly increased the compressive strength of the concrete throughout the day [12]. The compressive strength of concrete is governed by the strength and development of the CSH gel [77]. NA consumes Ca(OH)_2_ during the hydration reaction, which contributes to the production of the CSH gel [78]; therefore, adding NA in the appropriate amount increases the compressive strength [44]. The 28-day compressive strength of concrete was evaluated with the addition of 1, 2, and 3% NA, and the findings showed that the addition of 1% NA increased the compressive strength by 4.03%. The amount of NA added increased, as did the compressive strength; however, as NA reached 3%, the compressive improvement changed from 4.03% to 8% [44].

**Figure 4:**
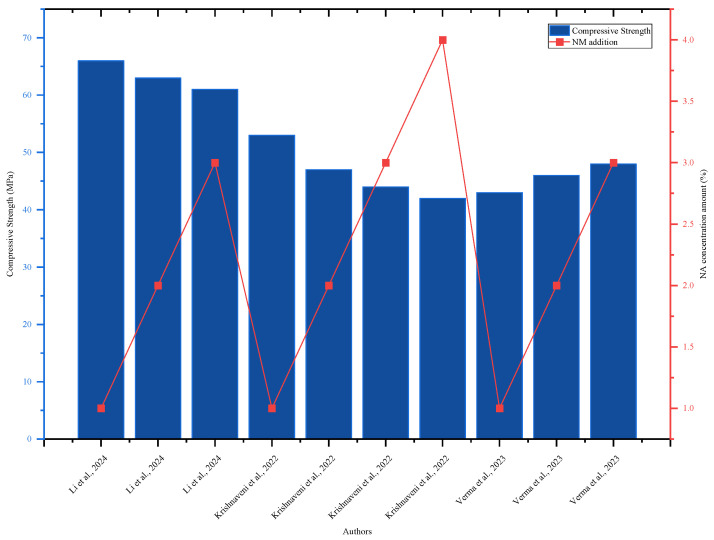
Effect of NA on 28-day compressive strength, highlighting strength improvement with increasing NA content by referring to different authors’ data [11,37,80].

Research by [45] investigated the impact of 0.15 and 0.25% NA on the incidence of fractures in high-performance concrete, maintaining a sand-to-cement ratio of 1.00 and a water-to-cement ratio of 0.5. NA contributes to the production of a strong concrete matrix because it acts as a bridge to C-S-H gel formation, providing the material with outstanding flexural strength. The inclusion of NA resulted in tensile strain values ranging from 0.005 to 0.006 mm/mm, which was lower than the control mix [45]. The findings of [79] show that the incorporation of NA has a considerable effect on the flexural strength of concrete. The flexural strength of the concrete improved as the NA concentration increased from 0.05 to 5% compared to the control mix. The highest flexural strength was 29.6 MPa after adding 3% NA, and it decreased afterwards. Adding 3.5–5% NA to concrete reduced its flexural strength compared to 3% NA, although it was still higher than the control mix. This indicates that increasing the amount of NA beyond the appropriate level could negatively impact flexural strength [79]. Nano-aluminum improved the strain-hardening properties of concrete, increasing its flexural and tensile strength [45,80]. The concrete’s compressive strength increased with the addition of NA until it reached the optimal level of 3%, as shown in Figure 4, and then dropped as the concentration rose. According to an assessment of concrete performance, the ideal NA addition is 3%, which corresponds to the highest concrete quality.

**Figure 5:**
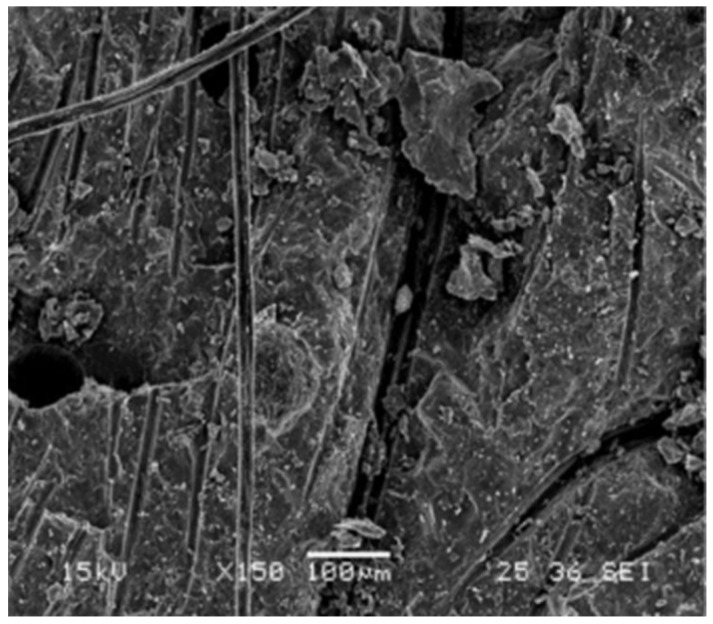
SEM image of NA’s synergistic impact on the concrete’s interior structure [46].

Referring to Figure 5, the strain-hardening behaviour was initiated by the inclusion of NA, which enhanced the matrix of the concrete’s internal structure close to the fiber connection that exists between NA and cement paste [46].

#### 3.2.3. Carbon Nanotube (CNT)

CNTs have a substantial influence on the mechanical strength of concrete up to the optimal level but increasing them above the optimum level reduces concrete strength [13,81]. The maximum compressive strength of 119.3 MPa was obtained with a 0.1% CNT cured at 20 ± 2 °C for 28 days, indicating that a 0.1% CNT increased compressive strength by 31.8% when compared to the control mix [13]. Carbon nanotubes improve the early-age hydration reaction because CNTs enhance the formation of a C-S-H gel [14,48]; due to this, the compressive strength was improved with the addition of CNTs [14]. Research by [48] found that adding a 0.5% CNT improved compressive strength at 7 days by 27.8% when compared to the reference mix and improved compressive strength at 28 days by 24.5%.

Research by [14] examined the impact of a CNT in the 0.05–0.15% range on the mechanical strength of concrete after it was cured for 7 and 28 days at a temperature of 20 ± 2 °C. The maximum compressive strength was determined at a 0.1% CNT addition on 7 and 28 days [14]. The addition of a 0.05% CNT improved compressive strength by 2.1 and 0.6% for 7 and 28 days, respectively. Similarly, a 0.15% CNT improved compressive strength by 2.3% and 1.9% for 7 and 28 days [14]. The compressive strength of concrete containing a 0.1% CNT increased by 18.21 and 28.23% after 7 and 28 days, respectively, compared to the reference mix [82].

**Figure 6:**
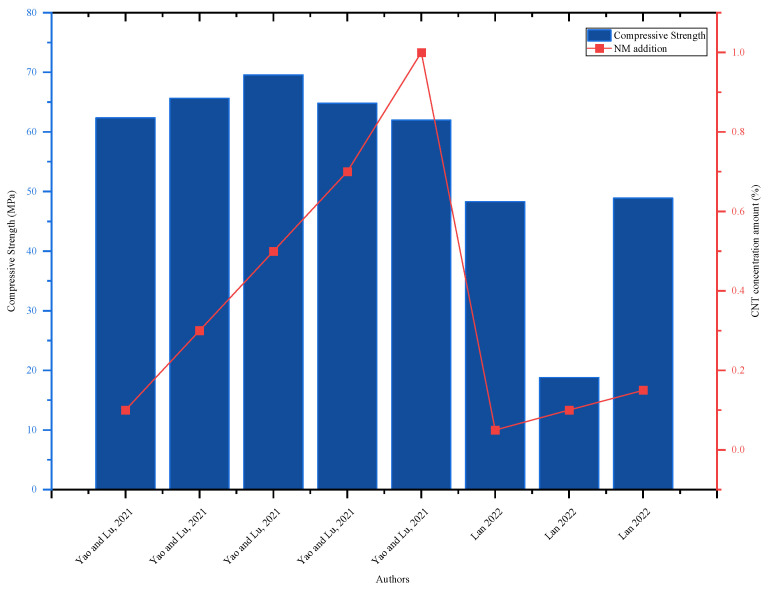
Effect of CNTs on 28-day compressive strength, highlighting strength improvement with increasing CNT content by referring to different authors’ data [14,48].

Study by [13] discovered that the addition of a CNT up to 0.1% improved the flexural strength of the concrete, but as the amount of the CNT was increased, the strength declined; the highest strength was reported at a 0.1% CNT. Similarly, the flexural strength of concrete rose by 13.68% with the addition of 0.15% addition [51], and 0.1% CNT increased the 28-day strength by 2.8% and the 7-day strength by 1.14% [82]. The CNT was designed to improve concrete tensile strength, and a 0.1% CNT improved split tensile strength by 6.17 and 5.17% for 7 and 28 days, respectively [82]; a 0.15% CNT improved 28-day strength by 29.35% [51]; and a 0.1% CNT improved by 17.8% on 7 days and 11.7% on 28 days [15]. As shown in Figure 6, [48] concluded that the concrete’s compressive strength increased significantly with the addition of a CNT up to 0.5% and subsequently decreased, but [14] experiment revealed that the addition of a CNT had divergent improvement. In this aspect, further investigation is needed to decide the optimal range of CNT content.

**Figure 7:**
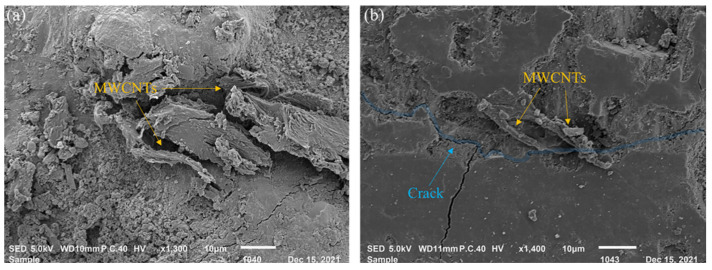
SEEM image OF CNT on filling (**a**) and bridging (**b**) [13].

As illustrated in Figure 7, the addition of CNTs filled the internal pore structure of concrete and made it thick, acting as a nano-reinforcement to bridge microcracks in concrete.

#### 3.2.4. Nano-Iron Oxide (NI)

Study by [15] investigated the influence of NI at 2, 3, and 4% on the mechanical characteristics of concrete and discovered that 3% NI was the ideal amount for the highest compressive strength. According to a compressive strength of 28 days, the inclusion of NI improved the strength of the concrete, while the compressive strength without NI was 36.52 MPa. A total of 2% of the NI increased to 51.68 MPa, 3% to 61.10 MPa, and 4% to 40.20 MPa [15]. Research by [30] discovered that the addition of NI increased the compressive strength, with the highest compressive strength achieved after 7 days of 11.8 MPa with the addition of 5% NI. In a study by [33], the compressive strength at 28 days was increased by 11.17 and 27.03%, respectively, with a replacement of cement of 1.5% and a 2.5% replacement of cement with NI. The addition of NI up to 2.5% improved the compressive strength of concrete at 3, 7, and 28 days, but the strength declined as the amount of NI increased. A total of 2.5% NI increased the compressive strength of the mortar by 13% after 3 days, 10% after 7 days, and 12% after 28 days [16]. The compressive strength decreased as the replacement NI increased; 3.5 and 5% replacement NI reduced the compressive strength at 28 days by 7.14 and 16.68%, respectively [33].

**Figure 8:**
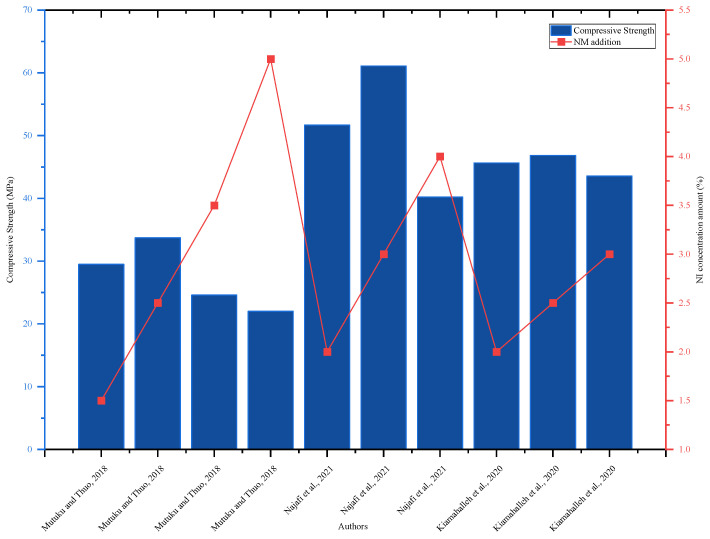
Effect of NI on 28-day compressive strength, highlighting strength improvement with increasing NI content by referring to different authors’ data [15,16,33].

The research by [15] investigated the effects of NI on the flexural strength of concrete and discovered that the addition of 0.3% NI increased the 28-day flexural strength of concrete by 58.2% as compared to the control mix. The flexural strength improved as the amount of NI increased to 2.5%, while when the amount of NI increased above 2.5%, the flexural strength decreased. The flexural strength of the mortar increased as the amount of NI increased to 2.5%; however, when the amount of NI increased above 2.5%, the flexural strength decreased [15]. The NI increased the flexural strength of the concrete with the best amount added, 2.5%. NI increased flexural strength by 2% after 28 days, 3% after 7 days, and 5% after 3 days. The addition of 1.5% NI to the concrete mix increased the tensile strength of the split on day 28 by 4.6%, but the addition of 2.5, 3.5, and 5% NI decreased the flexural strength on day 28 by 3.24%, 6.48%, and 13.43%, respectively [30]. As shown in Figure 8, the highest compressive strength was reached with 3% NI incorporation; adding NI over 3% reduced compressive strength. Based on performance evaluations and experimental limitations, the optimal quantity of NI addition was 3%.

**Figure 9:**
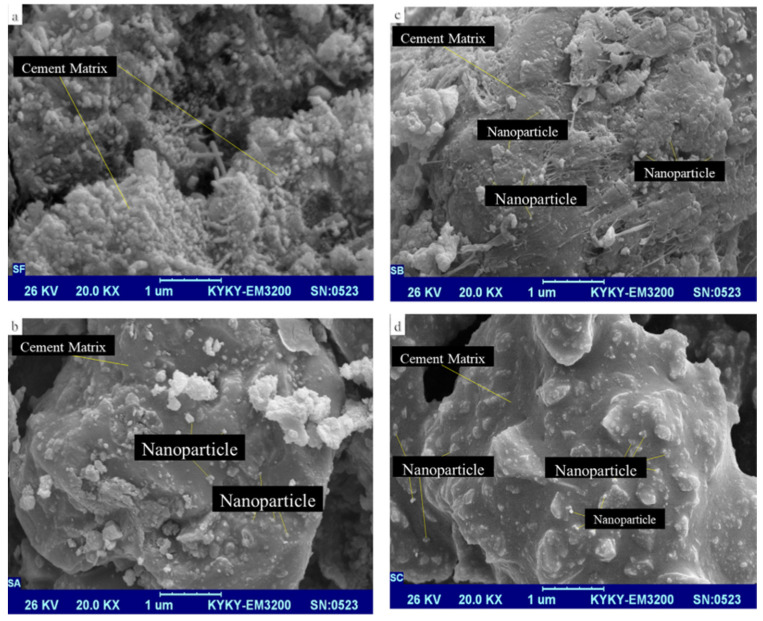
SEM image of concrete with (**a**) 0, (**b**) 2, (**c**) 4, (**d**), and 6% of NI [15].

As demonstrated in Figure 9, the interior concrete matrix was spongy and rough without NI. The addition of NI at dosages of 2, 4, and 6% resulted in a denser structure with fewer holes.

#### 3.2.5. Graphene Oxide (GO)

Referring to the findings of [83], the addition of graphene nanoplatelets (GPs) (0.5–2%) and GO (0.02–0.1%) becomes stronger and harder. Likewise, [84] found that with a 0.08% GO addition, at 28 days, the strength of concrete in the compression zone improved by 16.4% compared to the control mix. Similarly, the flexural strength increased by 27.1%. Furthermore, GO improved C-H crystallisation [84]. The addition of 0.03% GO improved the compressive strength CS by up to 46% after 7 days and by up to 28% at 28 days. Similarly, FS is enhanced by 28–50% on the 28th day of CS [17].

Ref. [34] investigated the effect of GO on the mechanical properties of concrete and found that the compressive, flexural, and split tensile strength improved significantly with the addition of GO in the range of 0.01–0.1%. The maximum strength was obtained with the addition of 0.1% GO, and the maximum 28-day compressive strength was 73.6 MPa, the flexural strength was 10.41 MPa, and the tensile strength of 6.7 MPa. The addition of GO increased the compressive strength of the concrete; the compressive strength of the concrete for 28 days with 0.08% GO addition was 47.26 MPa, which was 21% higher than the control mix, and at 28 and 56 days, the control mix exhibited a split tensile strength of 4.54 and 4.65 MPa with 0.08% GO addition, which was 12% higher than the control mix [18].

**Figure 10:**
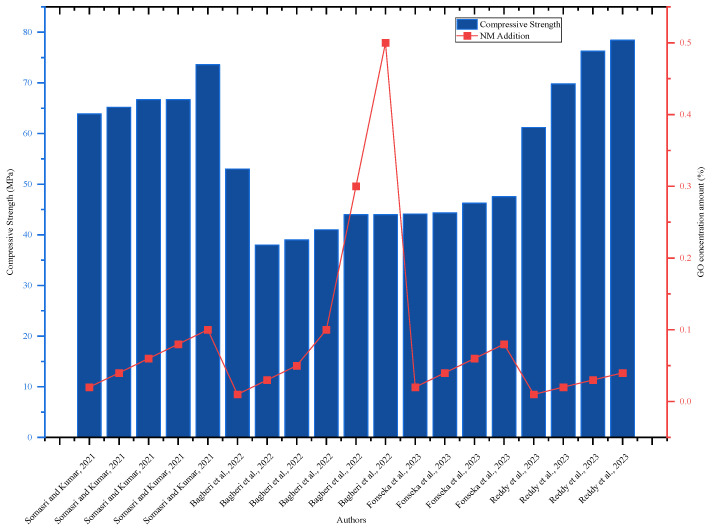
Effect of GO on 28-day compressive strength, highlighting strength improvement with increasing GO content by referring to different authors’ data [17,18,34,56].

The addition of GO significantly improved compressive strength; at 28 days, 0.1% GO improved compressive strength by 38.46% as compared to the control mix [56]. GO improved the flexural and tensile strength capacity of concrete at the early and long ages of 28 days, and 0.1% GO improved the 7- and 28-day flexural strength. At 7 days old, the flexural strength was 20.78% and the tensile strength was 17.05%; at 28 days, it improved by 14.87 and 12.07%, respectively [56]. The addition of GO significantly improved the overall performance of concrete because the GO material has a high strength capacity and dispersion tendency in water [85,86]. Figure 10 proves that the concrete’s compressive strength increased significantly with the addition of GO up to 0.1% but decreased as the GO level increased. In this regard, the best amount of GO addition is between 0.01 and 0.1%, which was established based on experimental limitations and performance evaluation.

**Figure 11:**
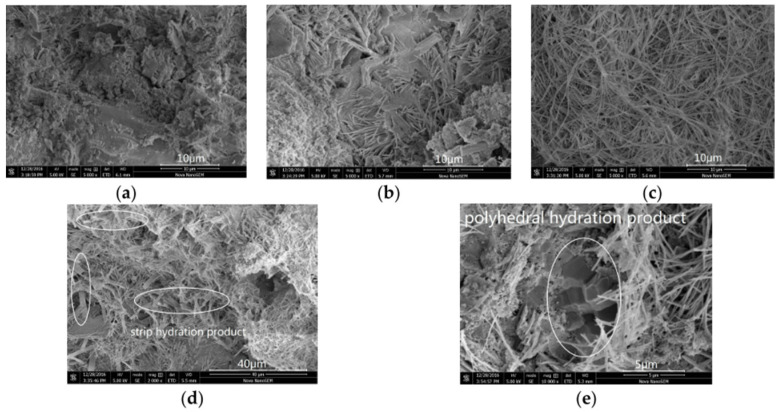
The cement hydration morphology effect with different GO addition after 28-day hydration reaction: (**a**) control, (**b**) 0.02%, (**c**) 0.04, (**d**) 0.06% and 0.08%, and (**e**) 0.08% GO, a polyhedral block shape product appeared and embedded in the [84].

Despite its huge surface area, reactivity, and high concentration of oxygen functional groups, GO modified the microstructure and enhanced the cement’s hydration process through the nucleation effect. The control mixture was more compacted, as Figure 11 shows. The addition of 0.02 and 0.04% GO enhanced the development of needle-like hydration products; 0.06% GO created a flat and long strip hydration structure, and 0.08% GO displayed a polyhedral block shape that bonded with other hydration products [84].

#### 3.2.6. Nano-Silica (NS)

The mechanical properties of concrete improved significantly as the concentration of NS increased [62]. According to [61], high-performance concrete that contains Electrical Arc Furnace (EAF) slag as an aggregate replacement with NS addition achieved a maximum compressive strength of 97.5 MPa when 1.5% NS was added to 100% aggregate replacement with EAF. The compressive strength of 70% granulated ground blast furnace slag (GGBS) blended concrete declined with NS but increased significantly when the GGBS proportion was reduced to 30%. At 7 days, adding 2% NS to the 30% GGBS concrete mixture increased compressive strength by 0.5% when compared to the control mixture. Comparably, 1% NS climbed by 8.1% [87]. The use of nanomaterials is essential for the development of sustainable concrete, as the addition of NS increases compressive strength at all ages. The highest compressive strength of 88.3 MPa at 90 days and 76.1 MPa at 28 days was demonstrated with 3% NS [64].

The addition of NS to a concrete mix including calcium carbide residue (CCR) as an alternative to cement significantly improved compressive strength. After 7 days of curing, a concrete mix containing 22.5% CCR and 1% NS improved its maximum compressive strength by 9.1% [88]. The addition of NS to concrete increased its compressive strength after 28 days; for concrete mixes that had NS added in 2, 4, and 6%, the corresponding improvements in compressive strength were 17, 24, and 16%, respectively. The concrete mix with 4% NS added had the highest compressive strength value out of all the mixes [89]. According to research by [64], adding NS to high-strength concrete increased the concrete’s split tensile strength. The corresponding split tensile strengths for NS incorporation in 1, 3, and 5% were 6.09, 6.46, and 6.04 MPa, respectively. Using a 3% NS addition, the maximum split tensile strength was achieved, exhibiting a 2% improvement over the reference mix [64]. The split tensile strength of concrete with calcium carbide residue (CCR) and NS added increased after seven and twenty-eight days. At 28 days, the split tensile strength of a mix containing 15% CCR and 1, 2, 3, and 4% NS addition increased by 21.3, 36, 30, 8, and 3%, respectively [88]. High-performance concrete’s split tensile strength improved with the addition of NS; when compared to the control mix, the split tensile strength increased by 13.0, 15.8, and 20.7%, respectively, with NS additions of 0.5, 1.5%, and 1.5% [61].

**Figure 12:**
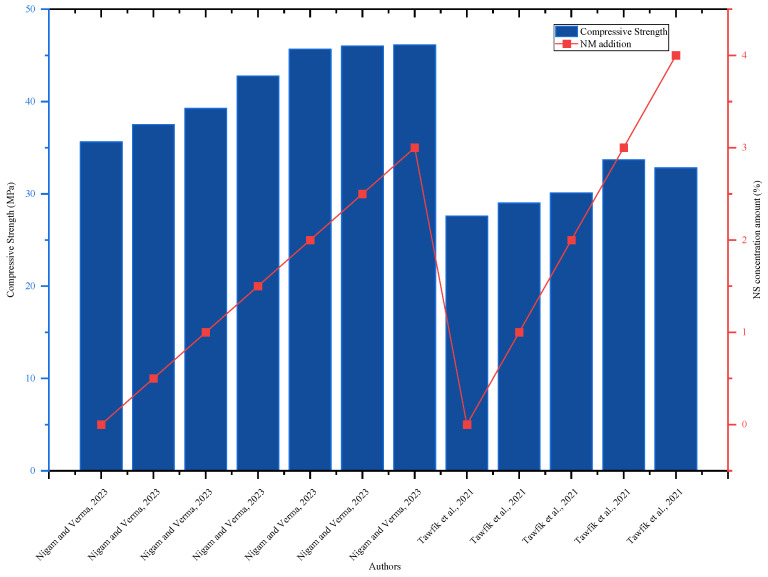
Effect of NS on 28-day compressive strength, highlighting strength improvement with increasing NS content by referring to different authors’ data [42,62].

The highest flexural strength of 7.57 MPa was achieved with 4% NS addition, which was 45% higher than the reference mix (5.24 MPa) [89]. NS significantly improved the flexural strength of the mixes containing CCR; for instance, concrete containing 22.5 CCR with the addition of 1, 2, and 3% NS improved the flexural strength by 4.6, 12.3, and 20% at 7 days and 8.2, 14.7, and 18.7% at 28 days as compared to the control mix [88]. The high reactivity of NS causes the formation of additional C-S-H gels, which densify the concrete microstructure and contribute significantly to mechanical strength enhancement [42,64,88]. As Figure 12 illustrates, the concrete’s compressive strength rose as NS was added until it reached the ideal quantity of 3%. According to a performance evaluation of the concrete, adding up to 3% NA is acceptable.

**Figure 13:**
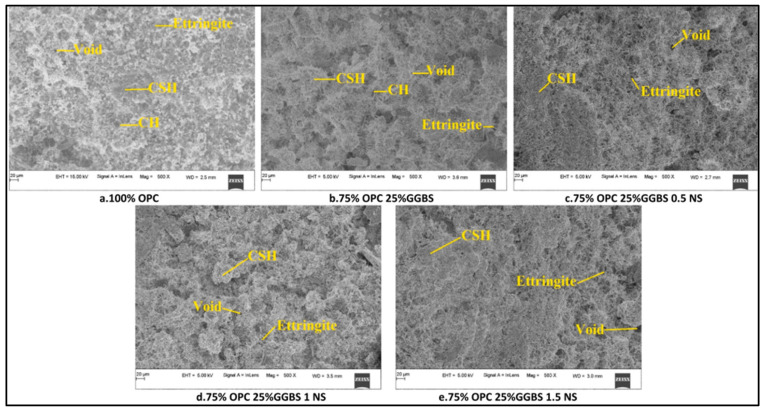
SEM image of cement pastes with NS addition after 28-day curing [61]. (**a**) 100% OPC, (**b**) 75% OPC 25% GGBS, (**c**) 75% OPC 25% GGBS 0.5 NS, (**d**) 75% OPC 25% GGBS 1 NS, (e) 75% OPC 25% GGBS 1.5 NS.

Figure 13 shows that a 1.5% NS addition with different cement replacement levels resulted in a thick microstructure of concrete. When 1 and 0.5% NS were introduced, undissolved binder components were found. The cement paste containing granulated ground blast furnace slag (GGBS) and 1.5% NS exhibited more C-S-H gel formation and fewer fractures.

#### 3.2.7. Nano-Titanium Oxide (NT)

Regarding NT’s impact on cementitious materials’ development of mechanical strength, it was discovered that adding NT to Portland cement (PC) pastes increased the pastes’ compressive, flexural, and split tensile strengths to an extent that varied with curing time and NT content [90,91]. The incorporation of NT improved the mechanical strength of concrete mixed with waste glass powder. The addition of NT at 1.825, 3.65, and 5.475% enhanced the compressive strength of concrete at 28 days by 3.38, 6.90, and 8.29%, respectively, and at 90 days by 12.78, 18.36%, and 19.18% [67]. The concrete’s compressive strength rose in parallel with the NT level due to improved interaction between NT and calcium hydroxide. Waste glass powder (WGP), being a pozzolanic material, tends to help in the development of compressive strength later, as seen by the increase in compressive strength on days 28 and 90 [67].

The impact of NT addition at 0.5, 1, 1.5, 2, and 2.5% on the compressive strength of concrete was investigated by [92]; the addition of 1% NT provided a maximum compressive strength of 62.33 MPa, which was 8% greater than the control mix. When the NT concentration exceeded the optimal (1%) level, the compressive strength decreased due to a reduction in accessible space for the production of C-S-H gel [92]. The control mix’s compressive strength after 28 days was tested to be 28.5 MPa, while adding 1% NT boosted it to 40.47 MPa. At 7, 28, 56, and 90 days of age, the highest compressive strength was achieved with the addition of 1.5% NT. A dose of NT at 1, 1.5, 2, and 2.5% increased the 7-day compressive strength of concrete by 2.15, 5.15, 10.67, and 6.67%, respectively; however, the 3% NT addition decreased the compressive strength by 3.34% [93]. The inclusion of NT at 2, 4, 6, 8, and 10% of the weight of fly ash increased the compressive strength of ultra-high-performance concrete. At 28 days of age, the compressive strength findings of 2, 4, 6, 8, and 10% NT addition were 173.4 m 188.9, 208.9, 205.8, and 201.2 MPa, respectively [66]. As a result, NT has a clear significant effect on the compressive strength development for both conventional and high-performance concrete [67,68,94] at all ages; however, the optimum amount varies from author to author. Future research will be needed to figure out the optimum quantity of NT.

Concrete flexural strength was improved by adding NT [67,68,94]. According to [67], adding 0.5, 1.5%, and 1.5% NT increased the 28-day flexural strength by 2.15, 6.47, and 8.03%, respectively. Compared to the control mix, the flexural strength increased by 10.57, 16.53, and 18.84%, respectively, after 90 days when 0.5, 1, and 1.5% NT were added [67]. As reported by [68], the maximum flexural strength of 4.24 MPa at 28 days, 3.08 MPa at 3 days, and 3.95 MPa at 7 days was attained with 1% NT addition.

**Figure 14:**
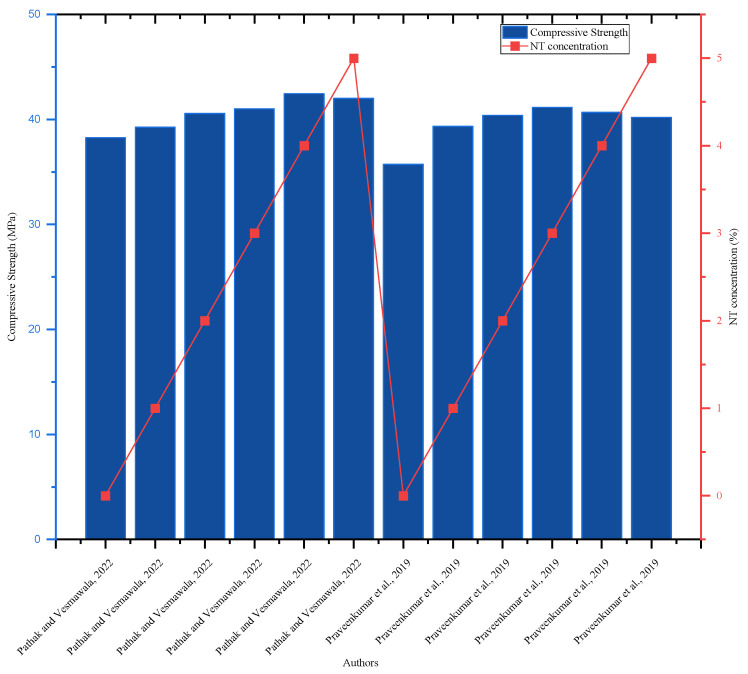
Effect of NT on 28-day compressive strength, highlighting strength improvement with increasing NT content by referring to different authors’ data [90,95].

Researchers have recently blended supplementary cementitious materials with nanoparticles to generate low-cost and green concrete. Ref. [95] investigated the effect of NT on fly ash-based concrete and found that the highest flexural strength of 4 MPa at 28 days was reached with 4% NT added to 20% fly ash blended concrete, which is equal to the control mix. In this aspect, using NT and fly ash reduced the amount of cement used while keeping the concrete’s quality [95]. The concrete’s tensile strength rose with NT addition from 0.5 to 1% and then decreased; for all curing times, the maximum strength was attained with 1% NT addition [68]. The 7-day splitting tensile strength of a concrete mix with NT concentrations of 0.5, 1.0, 1.5, 2, 2.5, and 3% was 2.3, 2.5, 2.8, 2.69, 2.65, and 2.48 MPa, respectively. The control mix had a split tensile strength of 2.2 MPa; hence, the addition of NT increased the split tensile strength of the concrete [93]. Referring to Figure 14, NT enhanced the mechanical properties of the concrete; however, the concentration amount differed from researcher to researcher; thus, more studies will be undertaken to establish the optimal quantity of NT addition.

**Figure 15:**
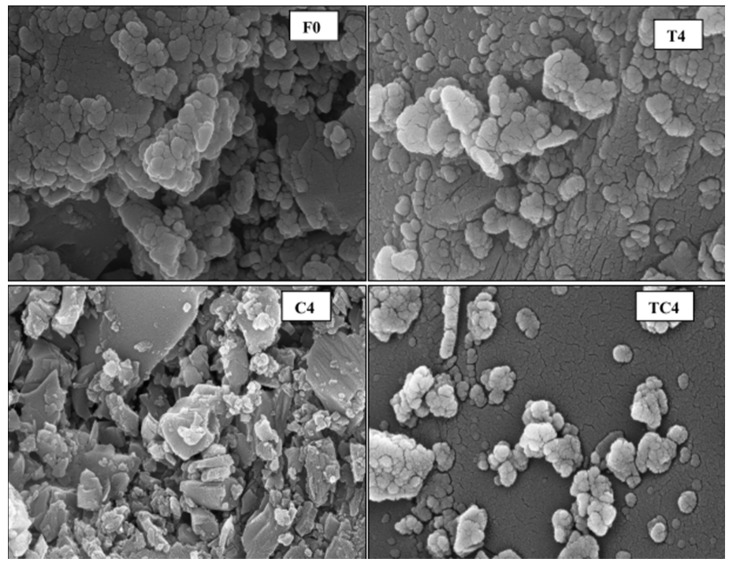
SEM image of concrete with NT addition [66].

Figure 15 shows the SEM image of the concrete with NT addition. Compared to the control mix (F0), the 2% NS addition reveals a uniform, dense microstructure.

### 3.3. Dispersion of Nanoparticles

Researchers investigated the effect of nanotechnology on the fresh and hardened properties of concrete (Section 2 and Section 3). However, one of the challenges was the dispersion of nanomaterials in water [41,94]. The techniques for the effective dispersion of nanomaterials in water were extensively explored, as shown in the section below. The dispersion of nanomaterials is critical for attaining the desired fresh and hardened properties [26,45] examined the dispersion of NA by physical dispersion using deionized water (DW) and superplasticizer (SP) admixture. Total quantities of 35 and 45 g NA were added to a container, followed by DW and SP; they were then stirred with a rod until they agglomerated readily and formed a slurry. The slurry’s composition was 94.6% DW, 3.8% NA, and 1.6% SP by weight [45]. Another researcher utilized an ultrasonication bath to disseminate the NA by ultrasonication techniques [12]. NCC was dispersed by sonication techniques using Sonics ultrasonic processing equipment, both with and without SP [35,96].

To disperse carbon nanotubes, conventional admixtures or polymers such as silane, acrylic particle dispersion, silica fume, and methyl-cellulose solution can be used [97], and this was a physical dispersion. Similarly, to create a homogenous mixed GO slurry, a sonication method of dispersion was also used [98,99]. Physical dispersion of GO can be achieved by blending it with 1% SP of the cement’s weight [100]. Another method is to mix GO with silica fume at 140 ± 5 rpm for 1 min [101]. Sonicating and dispersing NI in water for 10 min produced a homogeneous, dispersed solution, making NI dispersion easier than other nanomaterials [16]. Dispersing NS in a concrete mix is a challenging task. Sonication is a potential method for dispersing NS. According to [66], NT dispersion is crucial for significantly improving the mechanical properties of concrete. The use of mechanical string and surfactants allowed for the uniform dispersion of NT [66,94]. According to an overview of the existing literature, one of the challenges in using nanomaterials in the production of concrete is dispersing them in the concrete mix, which is one of the major barriers to their implementation. As a result, suitable dispersion methods have the potential to boost the effectiveness of nanomaterials in improving concrete qualities while simultaneously reducing the needed nanomaterial concentration by orders of magnitude. As previously stated, sonication is a potential dispersion method that readily disperses nanomaterials in the concrete mix both physically and mechanically; however, this is dependent on technological availability.

## 4. Benefits and Challenges of Nanomaterials in Sustainable Concrete

The production of concrete is considered unsustainable due to the depleting trend of the conventional ingredients [102]. Water must be available most of the time throughout the process; clinkers are used extensively to generate cement, and rocks serve to produce aggregates [77]. To make concrete more sustainable, researchers around the world advocate for the partial replacement of cement with added supplementary cementitious material (SCM). SCM has pozzolanic behaviour, and its development of strength takes a long time due to the secondary hydration reaction [103]. As the SCM is replaced in large amounts of cement, its mechanical strength decreases [104]. Similarly, the concrete faced low abrasion resistance [105], low resistance to carbonation, weak tensile strength, and the durability of concrete deteriorates as the quantity of SCM replacements increases [2]. To address this problem, nano material incorporation would be the best option. Concrete durability and strength are dependent on the interfacial transition zone strength, which mainly depends on the C-S-H gel formation [106,107]. Nano-reinforcement improves the development of the CSH gel and increases the tensile strength of concrete [5]. Due to this, the propagation of concrete cracks will be mitigated, and the strength and durability of concrete will be improved significantly.

The concrete structure’s strength and durability depend on the size of the structure, based on its design value. Structural engineering estimates the complexity of the structure by considering the strength of the concrete, span length, and economic considerations [5]. It is important to consider how to minimize the thickness of the concrete structure without hindering its strength and durability. The addition of nanomaterials significantly increases the performance of concrete [108], which means that it could minimize the size of the structure. When the size of the structures was minimized, the amount of concrete utilization also decreased. Concrete manufacturing consumes large amounts of cement and aggregate; the production of those materials and the overall process release carbon dioxide into the environment, and cement manufacturing releases 7–8% CO_2_ into the environment [109]. As a result, producing concrete utilizing nanoparticles becomes an excellent nanoengineering technique for the development of green concrete.

## 5. Conclusions

The effects of nanomaterials (NCC, NA, NI, NCT, GO, NS, and NT) on concrete properties were reviewed. Nanomaterials affect concrete by prolonging setting times and reducing workability and flowability, primarily due to their high surface area, which increases water demand. NA improves workability due to lower water absorption. Adding chemical additives and waste materials, like fly ash, can adjust workability and setting time. Nanomaterials enhance compressive, flexural, and tensile strength, and 4% NCC boosts early strength; NA, CNT, NI, and GO improve strength within specific ranges; GO is optimal for mechanical strength and material optimization; and NS and NT also boost strength, but NT requires further study. Nanomaterials enhance C-S-H gel formation, aiding concrete performance and sustainability. Despite the benefits, issues of dispersion, cost, scalability, and environmental impact need investigation. Further research on mix design, long-term performance, and fracture propagation with nanomaterials is required. The following recommendations for future research are presented.

Nanomaterials enhance the early compressive, flexural, and tensile strength of concrete. Further studies are needed on long-term mechanical strength beyond 90 days and the impact of nanoparticles on crack prevention. Research has mostly focused on compressive strength, neglecting the flexural and tensile aspects, which are significant. Future studies should investigate flexural strength, tensile strength, and elasticity in detail. Additionally, developing an efficient concrete mix design considering mechanical and fresh properties is unexplored. Future research should address this using machine learning. Nanomaterial dispersion affects concrete performance, necessitating an investigation of its effect on fresh and hardened properties. The combination of nanomaterials with cementitious materials and aggregate replacement also requires a thorough study, particularly regarding their impact on concrete rheology.

## Figures and Tables

**Figure 3:**
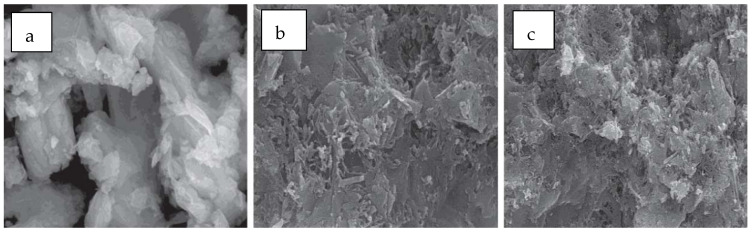
SEM image of NCC-modified concrete: (**a**) control, (**b**) cement blended with FA only, (**c**) sample with FA and NCC [10].

**Table 1:** Chemical compositions of nanomaterials.

Components	Content (%)				
	NCC	NA	NI	GO	NS
CaO	97.8	--	0.36	--	0.06
Fe_2_O_3_	0.02	0.009–0.012	88.31	--	0.08
MgO	0.5	--	2.22	0.51	0.21
Al_2_O_3_	--	99.0	2.67	0.49	7.39
SiO_2_	--	0.01–0.015	4.21	1.41	92.5
NaO_2_	--	0.35–0.45	0.04	--	0.02
K_2_O	--	--	0.02	--	0.04
TiO_2_	--	--	0.01	--	--
MnO	--	--	0.21	--	--
C	--	--	--	74.87	--
O	--	--	--	28.49	53.33
S	--	--	--	--	6.83
Reference	[32]	[12]	[33]	[34]	[31]

## Data Availability

The data will be available upon request from the corresponding author.

## Abbreviations

The following abbreviations are used in this manuscript:

NCC	Nano-calcium carbonate
NA	Nano-aluminum
NCT	Nano-carbon nanotube
GO	Graphene oxide
NS	Nano-silca
